# The effect of age on the outcome of esophageal cancer surgery

**DOI:** 10.4103/1817-1737.49415

**Published:** 2009

**Authors:** Abbas Alibakhshi, Ali Aminian, Rasoul Mirsharifi, Yosra Jahangiri, Habibollah Dashti, Faramarz Karimian

**Affiliations:** *Department of General Surgery, Tehran University of Medical Sciences, Tehran, Iran*

**Keywords:** Elderly, esophageal cancer, esophagectomy, morbidity, mortality

## Abstract

**BACKGROUND:**

Surgery is still the best way for treatment of esophageal cancer. The increase in life expectancy and the rising incidence of esophageal tumors have led to a great number of elderly candidates for complex surgery. The purpose of this study was to evaluate the effects of advanced age (70 years or more) on the surgical outcome of esophagectomy for esophageal cancer at a single high-volume center.

**MATERIALS AND METHODS:**

Between January 2000 and April 2006, 480 cases with esophageal cancer underwent esophagectomy in the referral cancer institute. One hundred sixty-five patients in the elderly group (70 years old or more) were compared with 315 patients in the younger group (< 70 years). All in-hospital morbidity and mortality were studied.

**RESULTS:**

The range of age was 38–84 years, with a mean of 58.7. The mean age of the elderly and younger groups was 74 and 53.2, respectively. In the younger group, 70 patients (22.2%) and in the elderly group, 39 patients (23.6%) were complicated (*P* 0.72).The most common complications in the two groups were pulmonary complications (9.8% in younger and 10.3% in elderly) (*P* 0.87). Rates of anastomotic leakage and cardiac complications were also similar between the two groups. Hospital mortality rates in younger and elderly patients were 2.8% and 3%, respectively. There was no significant difference between the two groups in morbidities and mortality (*P*-value > 0.05).

**CONCLUSIONS:**

With increased experience and care, the outcomes of esophagectomy in elderly patients are comparable to young patients. Advanced age alone is not a contraindication for esophagectomy.

The aging of the population and a longer life expectancy have led to more elderly patients with cancers being referred for treatment. For many of them, in particular for esophageal cancer, surgery remains the mainstay of treatment. Undoubtedly, esophagectomy is a major surgical procedure, with high postoperative morbidity and mortality rates.[1–3]

There is no established cut-off to define a patient as “elderly” in relation to surgery, but most studies available so far set the age limit at 70.[2,3]

Controversies exist about the effect of advanced age on the outcome of esophagectomy.[3] There is contradiction between older reports in which the risk of mortality after esophagectomy is strongly related to the patient's age and performance status, with worse long-term survival among elderly patients[4–6] and more recent studies confirming improvements in the results of esophagectomy in patients 70 years old and older (mostly as a result of advances in peri- operative care), with morbidity and mortality rates comparable to younger patients.[1–3,7–9]

The purpose of this study was to evaluate the effects of advanced age (70 years or more) on the surgical outcome of esophagectomy for esophageal cancer at a single high-volume center.

## Materials and Methods

Between January 2000 and April 2006, 480 cases with esophageal cancer underwent esophagectomy in the referral cancer institute. One hundred sixty-five patients in the elderly group (70 years old or more) were compared with 315 patients in the younger group (< 70 years). Tumors located in the cervical esophagus and the subcardia as well as administration of neoadjuvant therapy were exclusion criteria of this study. Pre-operative workup included medical history, physical examination, routine laboratory tests, chest X-ray, pulmonary function tests, and electrocardiogram. In selected cases, more cardiac evaluation (echocardiogram, exercise test or radionuclide scan) were also performed. Barium swallow, upper gastrointestinal endoscopy, and computed tomographic scans of chest and abdomen were routinely performed. All patients underwent the transhiatal Orringer technique for lower esophageal tumors or transthoracic esophagectomy (Ivor–Lewis or Mckeown procedures) for the mid- and upper-third of the esophagus. Alimentary tract reconstruction was performed preferably with the gastric pull-up technique. If stomach was unavailable, either a jejunal loop or the left colon was used. All patients were managed in the intensive care unit for the immediate postoperative period. Cervical anastomoses were monitored clinically, resuming oral diet on the fifth postoperative day. Contrast study was performed only for patients with intrathoracic anastomosis or in any cases with suspected leakage. All in-hospital morbidity and mortality were studied. Three categories of complications were considered in this study: Anastomotic leakage, pulmonary complications (pneumonia, aspiration, atelectasis, collapse, and respiratory failure), and cardiovascular complications (myocardial infarction, severe arrhythmia, heart failure, pulmonary edema, and pulmonary embolism). All data were collected retrospectively from patient's charts. Comparisons between two groups were performed using the χ^2^ test. A *P*-value of <0.05 was considered statistically significant.

## Results

The characteristics of 480 patients who underwent esophagectomy in the elderly and the younger groups are summarized in Table 1. The range of age was 38–84 years, with a mean of 58.7. The mean age of the elderly and the younger groups were 74 and 53.2, respectively. The male to female ratio was about 2:1. Squamous cell carcinoma constitutes 94% and adenocarcinoma constitutes 6% of the cases. Most of the patients were in stage III, followed by stage II. Most tumors were located in the lower part of the esophagus (55%). There were no significant differences in sex, histology, stage, location, and type of operation between the two groups.

**Table 1:** Characteristics of patients who underwent esophagectomy in the elderly and the younger groups

	<70 years (%)	≥70 years (%)	*P* value
No. of patients	315	165	
Mean age (years)	53.2	74	
Sex (male/female)	202/113 (64)/(36)	102/63 (62)/(38)	0.618
Histology			0.696
Squamous cell carcinoma	295 (93.6)	156 (94.5)	
Adenocarcinoma	20 (6.34)	9 (5.5)	
Stage			0.529
I	16 (5)	7 (4.25)	
II	81 (25.7)	39 (23.6)	
III	210 (66.7)	115 (69.7)	
IV	8 (2.5)	4 (2.4)	
Location			0.709
Upper thoracic	16 (5)	7 (4.2)	
Middle thoracic	122 (38.7)	70 (42.4)	
Lower thoracic	177 (56.3)	88 (53.3)	
Types of esophagectomy			0.741
Transthoracic	129 (41)	65 (39.3)	
Transhiatal	186 (59)	100 (60.7)	

Details of the postoperative complications in the two groups are given in Table 2. In the younger group, 70 patients (22.2%) and in the elderly group, 39 patients (23.6%) were complicated (*P*-value >0.05) [Table 2]. The most common complications in the two groups were pulmonary complications (9.8% in younger and 10.3% in elderly) (*P*-value >0.05). Rates of anastomotic leakage and cardiac complications were also similar between the two groups. Hospital mortality rates in the younger and elderly patients were 2.8% and 3%, respectively. There was no significant difference between the two groups in morbidities and mortality (*P*-value >0.05).

**Table 2:** Postoperative morbidity and mortality

	Younger group (%)	Elderly group (%)	*P* value
Complications	70 (22.2)	39 (23.6)	0.72
Leakage	25 (7.9)	12 (7.2)	0.79
Pulmonary	31 (9.8)	17 (10.3)	0.87
Pneumonia	10 (3.2)	5 (3)	
Aspiration	2 (0.6)	2 (1.2)	
Atelectasis	12 (3.8)	5 (3)	
Collapse	0	1 (0.6)	
Respiratory failure	7 (2.2)	4 (2.4)	
Cardiovascular	14 (4.4)	10 (6)	0.59
Myocardial infarction	7 (2.2)	4 (2.4)	
Severe arrhythmia	1 (0.3)	2 (1.2)	
Heart failure	1 (0.3)	0	
Pulmonary edema	1 (0.3)	0	
Pulmonary embolism	4 (1.2)	4 (2.4)	
Mortality (in-hospital)	9 (2.8)	5 (3)	0.70

## Discussion

Increase in the elderly population in the recent decades has made surgeons face the dilemma of whether to perform complex surgery on an elderly patient. The need for surgery for old patients is becoming a serious and crucial health problem. There have been advances in the management of many tumors recently but, for many of them, surgery remains the mainstay of treatment. Undoubtedly, esophagectomy is a major surgical procedure with high postoperative morbidity and mortality rates.[3] Old papers reported disappointing outcomes of esophagectomy in the elderly [Table 3].[4–6] Abunasra *et al*. analyzed the predictors for death after esophageal resection. In that study, advanced age, impaired pre-operative pulmonary status, and a tumor located high in the esophagus are associated with a significantly increased risk of postoperative death. They showed that the risk of operative death almost doubles for each 10-year increase in the age of the patient.[10] However, several recent retrospective studies have shown comparable morbidity, mortality, and survival rates between the elderly and the younger patients.[1–3,7–9] The idea that older age itself contraindicates major surgery such as esophagectomy seems to be a misconception, especially because of acceptable survival benefit for selected old patients.[3]

**Table 3:** Summary of some of the most important articles comparing esophagectomy for cancer between elderly and young patients*

Authors	Period	Age (years)	*N*	Respiratory complications (%)	Cardiac complications (%)	Anastomotic leakage (%)	Mortality (%)	Hospital stay (days)	Survival (%)
Thomas *et al.*[13]	1979-1994	≥70	56	17.9	3.6	10.7	10.7	19.8	
		<70	330	20.6	1.8	13.6	11.2	21.5	
Jougon *et al.*[14]	1980-1993	≥70	89	8	1.3	11.2	7.8	23.3	13.3
		<70	451				5.3	23	20.7
Poon *et al.*[4]	1982-1996	≥70	167	**39.5**	**37.1**	4.8	18		32
		<70	570	**28.1**	**20.5**	3.9	14.4		37
Ellis *et al.*[9]	1970-1997	≥70	147	4	6.4	3.4	5.3		26.8
		<70	358	3.5	4.8	6.4	2.4		24
Alexiou *et al.*[15]	1987-1997	≥80	36	19	**11.1**	5.6	5.6	13.5	21.6
		70–79	150	25	**7.3**	5.3	6.7	13	23.9
		<70	327	16	**2.1**	5.6	4.7	12	28.3
Ma *et al.*[16]	1990-2004	≥70	60	**43.2**	38	3.3	3.3		
		<70	1782	**28.1**	**20**	2	1.1		
Ruol *et al.*[3]	1992-2005	≥70	159	17	9.6	7.5	1.9		35.4
		<70	580	15.3	5	10.2	2.7		33.6
Internullo *et al.*[2]	1991-2006	≥76	108	37	19.4	2.8	7.4	15.5	35.7
		<75	1107						
This article	2000-2006	≥70	165	10.3	6	7.2	3		
		<70	315	9.8	4.4	7.9	2.8		

Statistically significant differences are shown in bold;

* Modified table from Internullo *et al.*[2]

Of course, careful pre-operative risk assessment is important in selecting patients for esophagectomy. Pulmonary complications were the frequent causes of morbidity among both younger and older patients, which emphasizes the importance of good pre-operative preparation, with aggressive chest physiotherapy before and after the operation, performed routinely by patients or preferably by a respiratory therapist. The rate of cardiovascular complications and leakage were also similar between the two groups in our study. We routinely performed hand-sewn cervical anastomosis, with an acceptable low risk of leakage. The main predictor of postoperative cardiovascular complications is the presence of pre-operative cardiac diseases. With precise pre-operative cardiac evaluation, the rate of operative morbidity and mortality are decreased.

The mortality rate in this study was about 3%, which is comparable to that in other studies.[1–3,7–9] The mortality did not differ significantly between our two age groups and there are several factors responsible for this result. First is the great experience of the cancer surgeons in our tertiary cancer institute. Advanced peri-operative care has also contributed to reduce the related morbidity and mortality.

One alarming issue in our report, which was derived from a developing country without any screening program, is the presence of only about 30% of the tumors in stage I and II in both groups, which may lead to more complications and lower survival. In comparison, in other studies, 40–70% of the tumors were diagnosed in low stages.[2,3,10]

The major limitation of this study is analysis of data of only the selected elderly population who were relatively fit for surgery. A large number of old patients did not undergo operation in comparison with the young group because of comorbidities. Another important issue is that our results come from a specialized high-volume cancer institute. Several reports showed that advanced procedures have lower complications in high-volume centers.[11,12] Thus, generalization of these results may not be valid in every center.

In conclusion, with increased experience and care, the outcome of esophagectomy in elderly patients is comparable to young patients. Advanced age alone is not a contraindication for esophagectomy. For selected patients with good operative risks, esophagectomy is indicated, regardless of age.[16]
